# Understanding the cellular architecture of Huntington’s disease

**DOI:** 10.7554/eLife.112225

**Published:** 2026-07-14

**Authors:** Dorian Pustina

**Affiliations:** 1 CHDI Management, Inc Princeton United States

**Keywords:** Huntington's, neurodegeneration, Neurite Density, Imaging, Human

## Abstract

A new diffusion MRI approach offers a glimpse of the anomalies of cellular architecture underlying basal ganglia degeneration in Huntington’s disease.

**Related research article** Ioakeimidis V, Palombo M, Casella C, Layland L, McNabb CB, Schubert R, Pallmann P, Busse ME, Drew CJ, Alusi S, Harrower T, Rosser AE, Metzler-Baddeley C. 2025. In vivo mapping of striatal neurodegeneration in Huntington’s disease with soma and neurite density imaging. *eLife* **14**:RP107661. doi: 10.7554/eLife.107661.

Modern neuroimaging has become remarkably effective at measuring how much brain tissue is lost during neurodegeneration. What remains much harder is determining what cellular changes produced that loss. Are neurons disappearing? Are glial cells proliferating? Is the extracellular space expanding? Conventional MRI can reveal the consequences of these processes but generally cannot distinguish among them.

Huntington’s disease provides a powerful test bed for methods that aim to bridge the gap between cellular pathology and non-invasive biomarkers. The condition is caused by a single genetic mutation, follows a relatively predictable biological course, and is characterized by progressive degeneration of two regions in the brain called the caudate and putamen, which are part of the striatum (Waldvogel et al., 2015).

The development of disease-modifying therapies is changing what is required from imaging biomarkers. Rather than simply documenting volume loss, biomarkers are increasingly expected to detect the earliest biological consequences of the mutant huntingtin gene long before clinical symptoms emerge, and to report on the processes that disease-modifying therapies are designed to alter (Scahill et al., 2020; Tabrizi et al., 2022a).

Now, in eLife, Claudia Metzler-Baddeley and colleagues at research institutes in the United Kingdom and Denmark – including Vasileios Ioakeimidis as first author – report on how MRI can move beyond measuring tissue loss to detect the microstructural changes that underlie it (Ioakeimidis et al., 2025). Rather than focusing on how much the striatum has degenerated in Huntington’s disease, they ask whether MRI can reveal changes in its cellular architecture in vivo.

Ioakeimidis et al. applied Soma and Neurite Density Imaging (SANDI), a diffusion MRI model that estimates MRI-derived indices of neuronal cell bodies, neurites and the extracellular compartment. Although these quantities are not direct histological measurements, previous studies suggest that they capture biologically meaningful aspects of tissue microstructure (Palombo et al., 2020; Barakovic et al., 2024).

The central finding is that several SANDI indices change in ways that mirror decades of neuropathological observations in Huntington’s disease. Reduced apparent soma density is consistent with the well-established loss of medium spiny neurons, whereas increases in extracellular signal fraction are compatible with a reduction of tissue density accompanying degeneration (Hedreen et al., 2024). These measures were also associated with motor impairment, disease burden, and regional atrophy, suggesting that they may complement existing volumetric biomarkers by providing information about the cellular processes driving striatal degeneration (Figure 1). For a disease in which neuronal loss has long been inferred rather than measured in vivo, this represents an important conceptual advance.

**Figure 1.**
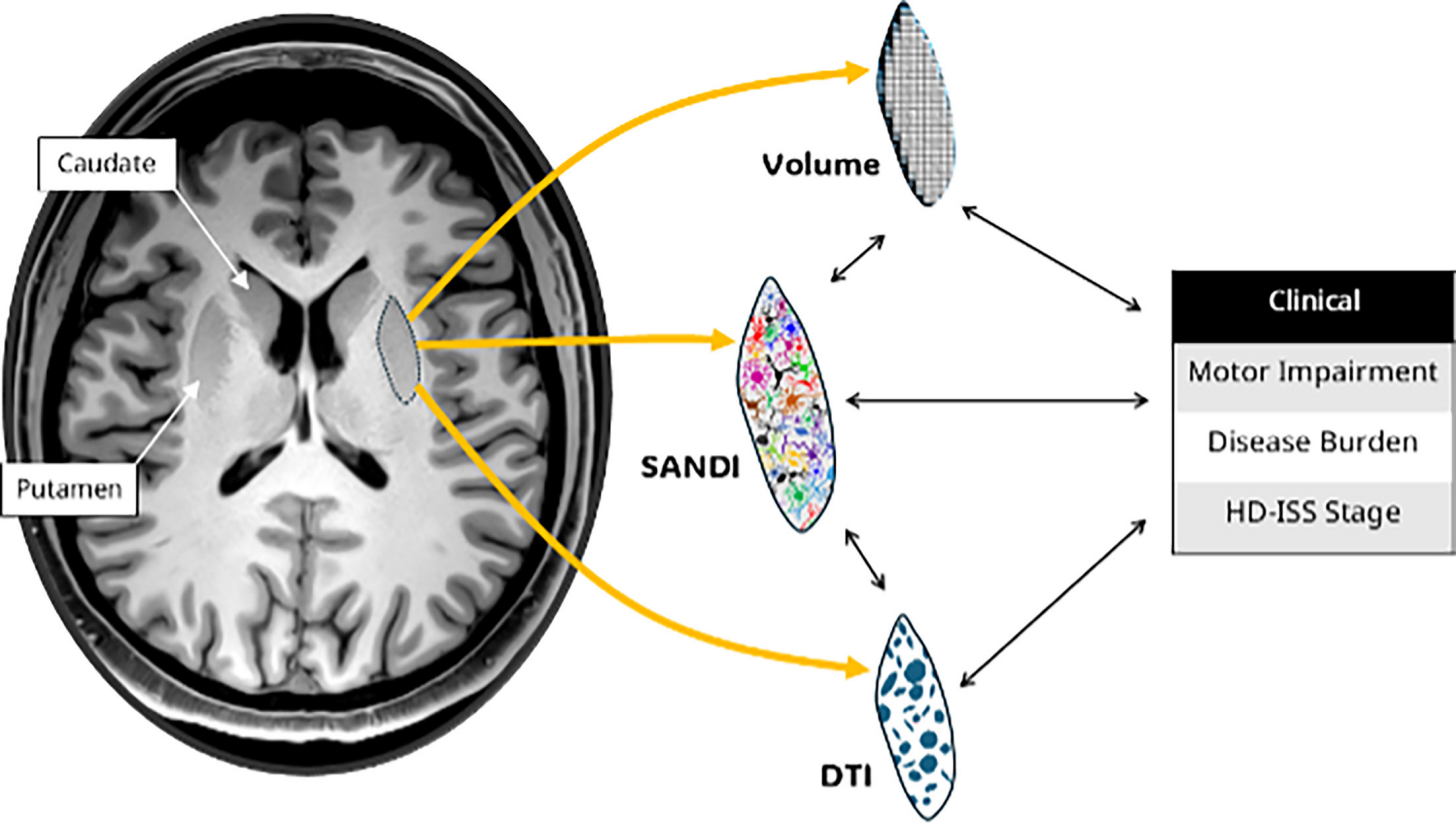
Overview of the study on soma and neurite density imaging (SANDI). Ioakeimidis et al. extracted SANDI measures from the subcortical nuclei, including the caudate and putamen, alongside volume and diffusion tensor morphometry (DTI) data, to investigate their relationships with clinical features of Huntington’s disease and with each other (black arrows).

For more than two decades, volumetric MRI has been one of the most robust imaging biomarkers in Huntington’s disease, playing a central role in natural-history studies and emerging as the only biological marker of progression in the Integrated Staging System (Tabrizi et al., 2022b; Kinnunen et al., 2021). Yet, volume is ultimately the cumulative consequence of many biological events. By attempting to separate changes related to cell bodies, neurites and extracellular space, the work of Ioakeimidis et al. points toward a new generation of biomarkers that may help explain why the striatum is shrinking rather than simply documenting that it has.

As with any emerging biomarker, important questions remain. The current study combines data from multiple cohorts with different phenotypic measurements, and longitudinal studies will ultimately determine whether SANDI can track disease progression more sensitively than established imaging biomarkers. Equally important will be demonstrating that comparable measurements can be obtained across MRI systems that are more widely available. Finally, while the observed changes are broadly consistent with known Huntington’s disease neuropathology, some findings, such as preserved neurite density despite lower soma density, highlight how much remains to be learned about the biological processes that underpin the disease.

Throughout the history of neuroscience, conceptual advances have often followed technological ones. By making MRI increasingly sensitive to features of tissue microstructure rather than simply tissue loss, the study of Ioakeimidis et al. paves the way for the Huntington’s disease community to begin understanding neuropathological processes using non-invasive methods. The newly identified alterations in soma size and soma density now call for studies across the full spectrum of Huntington’s disease progression, using larger cohorts, longitudinal follow-up, and deeper neuropathological validation, to determine whether these MRI-derived measures can mature into clinically useful biomarkers.
